# Abnormal behavior capture of video dynamic target based on 3D convolutional neural network

**DOI:** 10.3389/fnbot.2022.1017748

**Published:** 2022-10-26

**Authors:** Fei Chen

**Affiliations:** School of Intelligence Engineering, Shandong Management University, Jinan, China

**Keywords:** surveillance video, target behavior, dynamic capture, convolutional neural network, semantic algorithm

## Abstract

The use of computers to understand video content can accurately and quickly label various videos. Behavior recognition technology can help users filter the video by screening the content. However, this calculation mode, which is only sensitive to the features in a pixel neighborhood, cannot effectively extract cross-frame long-range video features. In addition, the common long-range dependency capture methods are based on pixel pairs, which contain less semantic information and cannot accurately model dependencies. Based on this, this paper generates semantic units with rich semantic information in the form of neighborhood pixel aggregation and proposes a multi-semantic long-range dependency capture algorithm to solve this problem, which makes the established dependency relationship more accurate. At the same time, this paper proposes an early dependency transfer technology to speed up the reasoning speed of the multi-semantic long-range dependency capture algorithm. By embedding the proposed algorithm into the original convolutional neural network, and conducting sufficient performance tests and evaluations on different data sets, it is shown that the proposed algorithm outperforms other current algorithms in terms of recognition accuracy and achieves the optimal recognition effect, which can effectively enhance the long-range dependency capture ability and temporal modeling ability of the convolutional network, and improve the quality of video feature representation.

## Introduction

Surveillance video is a type of time series data composed of a large number of continuous frames. The Strong Contextual Correlations between frames constitute the spatiotemporal structure of the video, which is also the essence of video dynamics (Li et al., 2018). The elements contained in the video will appear many times at different positions in different frames and will derive long temporal and spatial dependencies over time. However, due to the limitation that local operations (e.g., convolution operations) cannot incorporate information from a larger region into the computation process, these long-range dependencies require the superposition of multiple local operations (e.g., the superposition of multiple convolution layers) to be captured (Hu et al., 2018). This approach has two main shortcomings: on the one hand, this long-range dependency capture method is inefficient; on the other hand, it is difficult to ensure that the captured dependencies are sufficient and accurate. Due to the development of imaging technology in capturing depth information in real time, more and more work has begun to study the application of depth data captured by depth cameras to behavior recognition problems. Depth image information is not sensitive to illumination and can provide body shape information as well as motion-related information, which can be used to help distinguish between similar motions generated from a single view (Wang et al., 2018).

Huang et al. (2020) proposed behavior recognition by using depth sequence maps, which provides additional body shape information and motion information. In their proposed method, the depth map is projected onto three orthogonal Cartesian planes, and a motion map (DMM) is generated by accumulating the global motion of the entire video sequence to take advantage of the additional information provided by the depth map. Finally, the local features and shapes of DMM are described by the histogram of gradient orientation, and the HOG descriptors extracted from the depth motion maps of each projection view (front, top, side) are combined into DMM-HOG. Qiu et al. (2019) proposed a descriptor for describing motion information at the depth sensor, called the oriented 4D surface normal histogram (HON4D). The authors use a histogram to describe the depth sequence so that it can capture the distribution of surface normals in time, depth, and spatial coordinates.

Similar to the extended receptive field, which can greatly improve the image recognition ability based on a convolutional neural network, taking more pixels into account when modeling long-range dependence will also improve the accuracy of dependence. It was inspired by the graph convolutional network which aggregates messages at adjacent nodes. Behaviors are located and classified, thereby speeding up the entire detection process. The 3D CNN is used to obtain the spatiotemporal information of the video, and the 2D CNN is combined to obtain the accurate spatial features. Aiming at the problem that the capability of extracting the video spatiotemporal features by the shallow 3D CNN is insufficient, this paper proposes a dependency capture algorithm based on semantic units by aggregating adjacent pixels into Semantic Units. It is named Multi-semantic Long-range Dependencies Capturing (MLDC) algorithm to solve the problem of long-distance dependency of video frames. Through performance tests and resolution experiments on Kinetics and Something-Something V1 data sets, the algorithm is proved not only to significantly outperform pixel-based algorithms and some mainstream 2D/3D networks but also to introduce only a very small amount of computation.

The main innovations of this paper are:

(1) It transmits a feature graph to an MLDC algorithm, establishes a corresponding long-range dependency for each semantic unit until the network is finished, and further enhances the modeling capability of the long-range dependency.
(2) A real-time dynamic capture scheme of abnormal events in surveillance video is proposed, and a real-time detection system of abnormal events in surveillance video is designed.
(3) Feature map visualization results were generated by the MLDC algorithm. The effectiveness of the MLDC algorithm is demonstrated by comparing the feature maps of the original networks.

## Related work

### Semantic segmentation

Traditional image segmentation methods, in which researchers use mathematical knowledge to solve image problems at an early stage. Due to the immaturity of the early conditions and the imperfection of the system, robust and accurate image segmentation cannot be achieved. With the development and progress of deep learning technology, more and more methods based on deep learning have been proposed to solve problems. With the rapid development of computer technology and hardware, more and more image algorithms apply deep learning to the field of image segmentation. Attention mechanism is an idea borrowed from NLP (Du et al., 2018). The method has the following advantages: 1) the network can focus on important places and suppress unnecessary pollution information; and 2) the feature expression capability of the network is improved. Non-local is the first work of attention mechanism in the field of semantic segmentation. By calculating the relationship between each pixel in the feature map, it generates a huge attention matrix map and aggregates dense context information (Tran et al., 2019). The proposal of Non-local can capture the long-distance interconnection between feature maps, thus breaking the limitation of the local receptive field of the convolution kernel, making the context information of various ranges establish links, greatly improving the segmentation accuracy of the network, but it also makes the operation more complex (Luo and Yuille, 2019). Based on Non, a variety of attention mechanism algorithms have emerged.

The attention mechanism method makes the accuracy of the semantic segmentation algorithm to a higher level. With the increase of the number of convolution kernel channels, the reconstruction performance of the network is gradually improved. This is because the number of convolution kernel channels is directly related to the dimension of the feature map, and too few channels will lead to a low dimension of the feature map. It is difficult for the network to learn useful information from low-dimensional features, which leads to the lack of fitting ability of the model and the low quality of the reconstructed video. Thereby capturing the temporal characteristics of the video. The feature calculation process on the *j* feature maps of the *i*-th layer of the 3D convolution defines:


(1) $$\begin{array}{l} v_{ij}^{xyz}=\tan b_{ij}+\underset{m}{\sum }\overset{p_{i-1}}{\underset{p=0}{\sum }}\overset{q_{i-1}}{\underset{q=0}{\sum }}\overset{r_{i-1}}{\underset{r=0}{\sum }}w_{ij}^{\;pqr} \end{array}$$

where *p, q* is the length and width of the 3D convolution kernel, *r*_*i*_ is the number of convolution kernels in the timing dimension, and $W_{ij}^{pqr}$ is the weight value of the convolution kernel connected with the *m*th feature map in the previous layer at the position *p, q, r*.

### Representation method based on dependency capture

Wang et al. (2018) proposed non-local neural networks. The non-local network effectively improves the quality of video feature extraction by adding global information to the feature map. Qiu et al. designed the Global to Local Diffusion module and the Local to Global Diffusion module to effectively transfer the dependency between the shallow and deep layers of the network (Feichtenhofer et al., 2019). The Corresponding Proposal Network (CPNet) proposed by Liu et al. (2019) interprets video as a dense Point Cloud. When modeling temporal information, the CPNet regards semantically similar Spatio-temporal points as Correspondence Proposals, and replaces all pixels in Non-Local with *K* proposal points. By learning the temporal information between the corresponding proposals, the CPNet improves the efficiency of mining spatiotemporal information (Jiang et al., 2019).

## Multi-semantic long-range dependency capture algorithm

The multi-semantic long-range dependency capture algorithm mainly consists of three parts, namely, pixel aggregation, correlation operation and dependency establishment. Pixel aggregation mainly refers to the aggregation of pixels and their surrounding pixels to form semantic units with strong semantic information. The relevance operation is mainly used to model the relevance between semantic units (Zhou et al., 2018). Finally, dependency establishment refers to determining another semantic unit that is most relevant to each semantic unit according to the result of the relevance operation.

### Algorithm description

(1) Pixel polymerization

The goal of a graph convolutional network is to learn a mapping that aggregates nodes and their neighbors. Similarly, the MLDC algorithm uses this approach for pixel aggregation (Lin et al., 2019). First, a semantic unit u is defined as a set of pixel features:


(2) $$\begin{array}{l} u\;=\;\left\{x_{1},x_{2},\cdots \;,x_{n}\right\} \end{array}$$

where *n* denotes the number of pixels in the semantic unit *u* and $x_{i}\in {\mathbb{R}}^{C}$ denotes a pixel with *C* channels.

There are two important principles that need to be followed when doing pixel aggregation:

1. The semantic unit must cover all pixels;
2. Each pixel can only be aggregated into one semantic unit.

**Figure 1:**
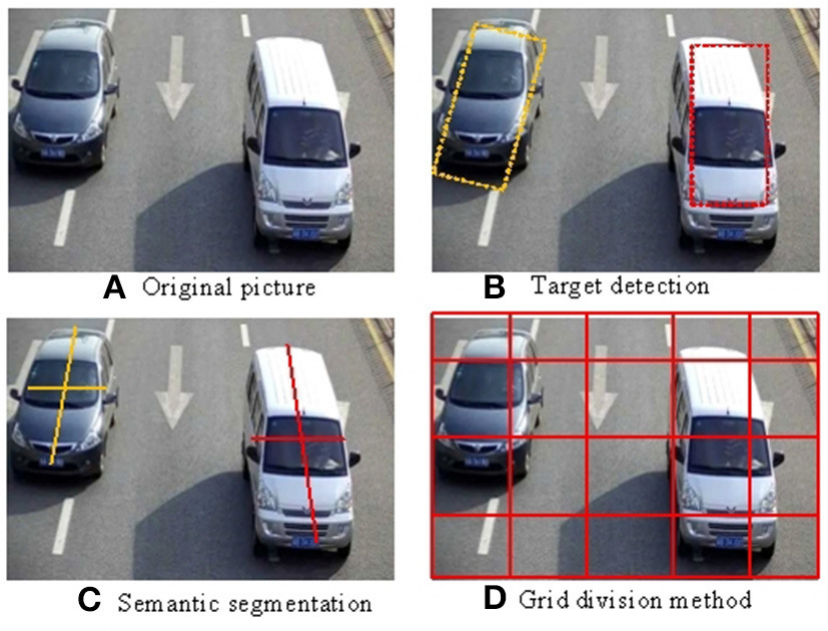
**(A–D)** Pixel aggregation method.

There are many ways to satisfy the above conditions. For example, the results of object detection (Figure 1A) or semantic segmentation (Figure 1C) are used as the basis for aggregation, which can only discriminate the frame of moving objects, ignoring part of the frame content. In addition, some clustering algorithms, such as K-means, can also meet the above requirements (Cao et al., 2019) for example, the object identified in (Figure 1C) can establish the target coordinates. Among these methods, the grid-based aggregation algorithm (Figure 1D) is the simplest but also quite effective in establishing the regional grid screening effect.

Given a video frame *f* ∈ ℝ^*C*×*H*×*W*^, define a mesh partition with mesh size s as a mapping χ :


(3) $$\begin{array}{l} \chi :{\mathbb{R}}^{C\times H\times W}\to {\mathbb{R}}^{\frac{H}{s}\times \frac{W}{s}\times s\times s} \end{array}$$

Each semantic unit is *u* ∈ ℝ^*C*×*s*×*s*^. The pixel aggregation methods used in the rest of this paper are all based on grid division (Qiu et al., 2019) in order to compute the core of the concise highlighting method.

(2) Correlation operation of semantic unit

A common way to describe correlation is to use the Dot Product operation:


(4) $$\begin{array}{l} f\left(x_{i},x_{j}\right)=x_{i}^{T}x_{j} \end{array}$$

where $x_{i},x_{j}\in {\mathbb{R}}^{C}$ is the pixel feature and *C* is the feature dimension (Liu et al., 2019). Given two semantic units *u*_*i*_, *u*_*j*_, the correlation between the two semantic units can be described as:


(5) $$\begin{array}{l} F\left(u_{i},u_{j}\right)=\overset{n}{\underset{k}{\sum }}f\left(u_{i}\left(k\right),u_{j}\left(k\right)\right)=\overset{n}{\underset{k}{\sum }}u_{i}{\left(k\right)}^{T}u_{j}\left(k\right) \end{array}$$

where *u*_*i*_(*k*) denotes the *k*th pixel of *u*_*i*_ in the semantic unit.

Further, for pixel aggregation based on a grid size *s*, equation (5) can be rewritten in the form:


(6) $$\begin{array}{l} F(u_{i},u_{j})=\sum_{h}^{s}\sum_{w}^{s}f(u_{i}(h,w),u_{j}(h,w))\\ \;=\sum_{h}^{s}\sum_{w}^{s}u_{i}{\left(h,w\right)}^{T}u_{j}(h,w) \end{array}$$

where *u*(*h, w*) represents the pixel with coordinate (*h, w*) in the semantic unit *u* (with the upper left corner of the semantic unit as the origin; Wang et al., 2018).

(3) Dependency established

This section describes in detail the process by which the MLDC algorithm models long-range dependencies in a multi-semantic manner. Formally, given a video *V* ∈ ℝ^*T*×*C*×*H*×*W*^ with T frames, where*C*, *H*, *W* are the number of A channels, the video height, and the width of the video, respectively (Kay et al., 2017). The aggregated video tensor size is $V\in {\mathbb{R}}^{T\times C\times \frac{H}{s}\times \frac{W}{s}\times s\times s}$.

Each semantic unit *u* ∈ ℝ^*C*×*s*×*s*^ finds its most relevant other semantic unit in the video *V* as a dependency (Ghiasi et al., 2021). The *T* video frames are two-dimensional convolution operations of the input, that is:


(7) $$\begin{array}{l} u^{corr}=2dConv\;\left(V,u\right) \end{array}$$

where *u*^*corr*^ ∈ ℝ^*T*×1×(*H*+1−*s*)×(*W*+1−*s*)^ contains the correlation value of the semantic unit u with each possible position in the video *V*. Then, the dependency of *u* is the video region *R*^*u*^ ∈ ℝ^*C*×*s*×*s*^ with which the maximum correlation value is generated. *R*^*u*^ is regarded as a dependent term of u (Li et al., 2019).

The dependency of each semantic unit has been established. Finally, all the dependencies are spliced into a tensor *R*^*v*^ ∈ ℝ^*T*×*C*×*H*×*W*^ of the same size as the input, and the output of the module is expressed as:


(8) $$\begin{array}{l} V_{out}=V+BN\left(R^{v}\right) \end{array}$$

where *BN*(▪) represents the 2D Batch Normalization operation.

### Module structure

**Figure 2:**
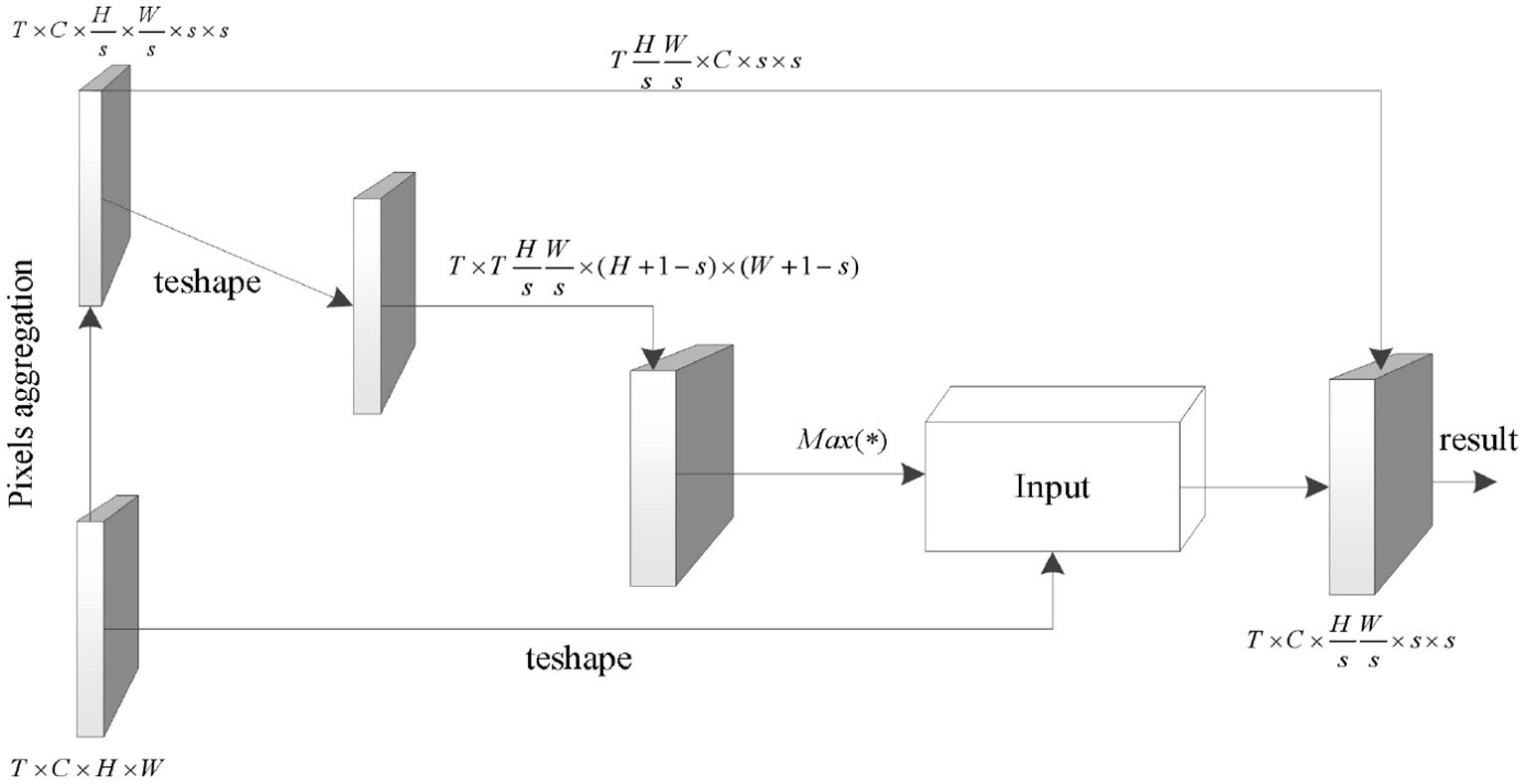
Structure of MLDC algorithm.

Figure 2 shows in detail how to combine all the parts included in the MLDC algorithm into a whole.

First, the pixel aggregation portion corresponds to transforming the input tensor *x* ∈ ℝ^*T*×*C*×*H*×*W*^ to $x\in {\mathbb{R}}^{T\times C\times \frac{H}{s}\times \frac{W}{s}\times s\times s}$. Then, in order to make full use of the relationship between the correlation calculation and the convolution operation obtained by the formula (3–5) and simplify the implementation difficulty of the correlation calculation, the input is converted to $x\in {\mathbb{R}}^{T\frac{H}{s}\frac{W}{s}\times C\times s\times s}$. The input is represented under this tensor size as a convolution kernel (Nunez et al., 2018) with the number of output channels *T* × *H* × *W*/*s*^2^, the number of input channels *C*, and the convolution size *s*. This convolution kernel is then convolved with the original form of the input to obtain a tensor $corre\in {\mathbb{R}}^{T\times T\frac{H}{s}\frac{W}{s}\times \left(H+1-s\right)\times \left(W+1-s\right)}$. This tensor preserves the relevance of each semantic unit to each region of the original input. By executing the *Max*(·) function, the coordinates of the region with the maximum correlation coefficient with each semantic unit are obtained, and finally the original pixel value of the coordinate region is added to its feature as the dependency of the corresponding semantic unit (Carreira and Zisserman, 2017).

### Overall network architecture

The TSN network divides each video into *K* equal-length timing intervals, and samples one frame from each timing interval to form an output sequence. The output of the TSN network is the average value of the output of the 2D convolutional network on each frame (Yu et al., 2021).

Given a video *V*, first extract the video frames of the video, and divide the video frames into *K* segments {*S*_1_, *S*_2_, ⋯ , *S*_*K*_}. Then, the modeling of the input sequence by the temporal segmentation network is expressed as:


(9) $$TSN(T_{1},T_{2},\cdots ,T_{K})=H(G(F(T_{1};W),F(T_{2};W),\cdots ,F(T_{K};W)))$$

where (*T*_1_, *T*_2_, ⋯ , *T*_*K*_) is a set of input sequences and each *T*_*K*_ is represented as a random sample of the timing interval *S*_*k*_. *F*(*T*_*K*_; *W*) represents a convolution operation on the input sequence, with a convolution kernel *W*. *G*(·) represents a fusion function, which is used to fuse the decision results of multiple sequences (the fusion method in the original text is average fusion) to obtain an output containing the probability distribution of the sample category (Teboulbi et al., 2021). Finally, *H*(·) stands for the *softmax*(·) function used to transform the output into a class probability distribution (Varol et al., 2017). In this paper, the Cross-entropy loss function is used to train the network. Finally, the optimization goal of the network is as follows:


(10) $$\begin{array}{l} L\left(y,G\right)=-\overset{C}{\underset{i=1}{\sum }}y_{i}\left(G_{i}-\log\overset{C}{\underset{j=1}{\sum }}\exp G_{j}\right) \end{array}$$

where *C* represents the number of categories. *y*_*i*_ denotes a referential function. *y*_*i*_ = 1 when *y* = *i* and *y*_*i*_ = 0 otherwise.

The sigmoid activation function is used to map to a value of 0 to 1, which determines whether to retain the information. When the threshold is larger, it means that the information is more important, otherwise, it means that the information should be forgotten.

**Figure 3:**
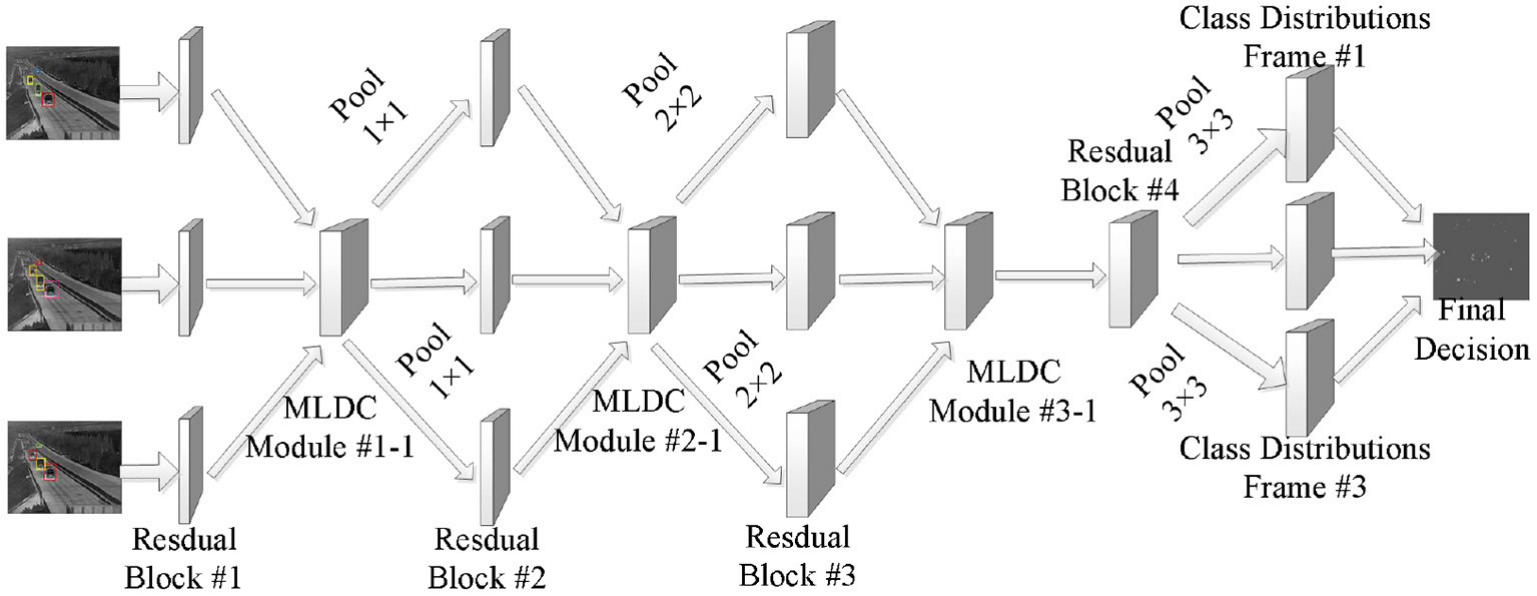
Overall framework of the network.

All MLDC algorithms in this paper are embedded outside the Residual Block of the backbone network. The network architecture after the MLDC algorithm is embedded into the TSN is shown in Figure 3.

The convolutional layer determines the output by computing the scalar product between the neuron weights and the region to which the input is connected (Wang and Gupta, 2018). The rectified linear unit (ReLU) applies the “sigmoid” and “elementwise” activation functions to the output produced by the previous layer. To explore the ability of MLDC algorithm in time series modeling, the time series transformation module (Temporal Shift Module) and MLDC algorithm are integrated into the traditional convolutional neural network, which can effectively improve the time series modeling ability of the network (Bouaafia et al., 2021).

### Operation efficiency analysis and optimization

**Figure 4:**
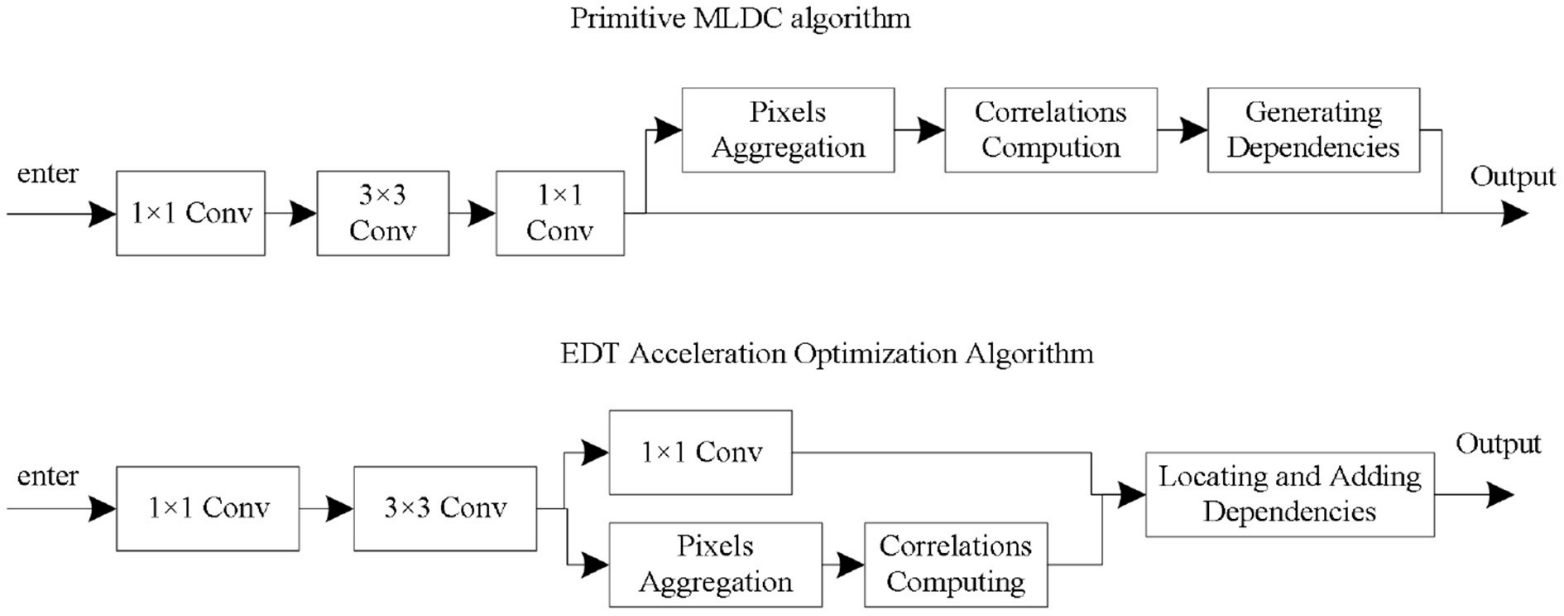
Technical optimization process.

Conventional dimensionality reduction methods (such as using an 1 × 1-convolution after each residual block) are not feasible. To solve this problem, this paper proposes an Early Dependencies Transferring (EDT) technique. Considering the low feature dimension in the residual block, the pixel aggregation and correlation calculation are partially moved into the residual block (Wang et al., 2018). Then, the coordinates of the most relevant regions of each semantic unit are transmitted to the outside of the residual block for modeling long-range dependencies. The flow chart of the EDT technique is shown in Figure 4.

In the EDT technique, the input data first goes through three convolution operations in the original residual module, namely convolution 1 × 1, convolution 3 × 3, convolution 1 × 1, and a residual concatenation operation. The feature map obtained after the completion of the second convolution requires pixel aggregation and correlation calculation operations, and the determined dependencies are directly transferred to the results outside the residual module (Seifeddine et al., 2020). Embed 1 EDT-optimized MLDC, which is the MLDCEDT. It introduces only <4% additional FLOPs. Embedding10 MLDCEDTs only improves FLOPs by an additional 14% and only slightly reduces classification accuracy (<0.5%).

## Experimental analysis

### Experimental data set

(1) Kinetics-400 data set is a video data set released by DeepMind in 2017. This is a high-quality, very popular, and very challenging large-scale video understanding data set. Among them, 234,619 samples were used for training and 19,761 samples were used for testing. This paper mainly uses this data set to test the performance of the MLDC algorithm and the digestion experiment (Cai and Vasconcelos, 2018).

(2) The Something-Something V1 dataset (Ssthv1) is a challenging dataset for fine-grained Action Recognition. The data set was collected and published by the TwentyBN platform in 2020. There are 174 classes and 108,499 samples in the Ssthv1 dataset. Among them, there are 86,017 training samples, 11,522 validation samples and 10,960 test samples (Kozlov et al., 2020).

Fusion reconstruction network training phase:

Input: learning rate α , minimum batch size *m*, maximum number of iterations *t*_max_.

Process.

1. Initialize network parameters and iterations.
2. while *t* < *t*_max_do.
3. Select *m* training videos (*x*_1_, *x*_2_, ⋯ , *x*_*m*_). Compute the corresponding compressive measurements *Y*_*i*_ = *x*_*i*_ + *n, i* = 1, 2, ⋯, *m*.
4. Calculate the compression measurement base reconstruction $x_{i}^{\prime }$ of *Y*_*i*_.
5. Use Adam method to update the parameters ϖ in the network.
6. *g*_ϖ_ ← *a* · *Adam*(ϖ, *g*_ϖ_).
7. Increase number of iterations *t* = *t* + 1.
8. end while.

Output: Trained network parameters ϖ.

### Experimental results and analysis

This paper demonstrates the performance of the MLDC algorithm on two large-scale video understanding datasets, Kinetics-400 and Something-Something V1. Among them, on the Kinetics dataset, a series of detailed digestion experiments are carried out to explore the optimal structure of the MLDC algorithm on the ResNet network (Seifeddine et al., 2022). Then, by comparing the optimal MLDC network structure with the current optimal algorithm, it is proved that the MLDC algorithm proposed in this paper not only outperforms the current optimal algorithm in recognition accuracy but also has great advantages in the total number of parameters and computational efficiency (Zoph et al., 2020).

### Digestion experiments on knietics-400 data set

(1) Embedding position of MLDC algorithm

Table 1 shows the different results produced by embedding the MLDC algorithm into different residual blocks.

**Table 1:** Experimental results of embedding MLDC modules at different locations of ResNet network.

**Layer**	**Top-1 (%)**	**Top-5 (%)**
Baseline	69.5	89.7
Res2	**72.7**	90.1
Res3	72.5	90.2
Res4	71.6	**90.6**
Res5	71.9	89.5

The bold values in each column are all the numbers with the largest percentages.

Adding a separate MLDC algorithm can effectively improve the network performance and enhance the network dependency capture ability. In addition, by comparing its impact in different layers, we can see that the MLDC algorithm will produce better results when it is embedded in shallow layers (for example, res2, res3). For example, embedding an MLDC algorithm into res3 results in a 2.5% performance improvement compared to the baseline performance. However, embedding in res5 will only result in a 1.1% performance improvement.

(2) Embedding MLDC multiple times

By equipping the backbone network with more MLDC algorithms, dependencies and related information can be propagated deeper into the network, thereby enhancing the ability of the network to model long-range dependencies and improving network performance. 1 MLDC embeds the MLDC algorithms with the number of 1, 2, 1 into the res3 layer, res4 layer, res5 layer respectively, and embeds the MLDC algorithms with the number of 2, 3, 3, 2 into the 5 MLDC, 10 MLDC respectively. The results are shown in Table 2.

**Table 2:** Experimental results of executing multiple MLDC algorithms in ResNet network.

**Number**	**Top-1 (%)**	**Top-5 (%)**
Baseline	69.9	89.0
1 MLDC	72.4	90.3
5 MLDC	73.0	91.0
10 MLDC	**73.2**	**91.1**

The bold values in each column are all the numbers with the largest percentages.

(3) Use longer input sequences

This section explores the performance of the network when longer input sequences are used. By expanding the input from 5 frames to 8 frames and 16 frames respectively, the results are shown in Table 3.

**Table 3:** Experimental results obtained using video frames of different timing lengths as input.

**# Frame**	**Top-1 (%)**	**Top-5 (%)**
5	73.2	91.1
8	74.1	91.6
10	**75.7**	**92.2**

The bold values in each column are all the numbers with the largest percentages.

Experiments show that the network has a very strong ability to model long sequences, which proves the effectiveness of MLDC algorithm in time series data modeling (Choi et al., 2020).

(4) Comparison of computational efficiency

This section compares the computational efficiency of the MLDC algorithm with some representative 2D algorithms (e.g., TSN, *R* (2 + 1) *D*) and 3D algorithms (e.g., I3D, S3D-G). The experimental results are shown in Table 4.

**Table 4:** Experimental results of embedding MLDC modules at different locations of ResNet network.

**Method**	**FLOPs × views**	**Top-1 (%)**	**Top-5 (%)**
TSN_8f_ (our impl.)	33G × 10	69.8	89.4
R (2+1) D	152G × 10	74.6	91.4
S3D-G	71.4G × 10	74.4	**93.7**
I3D	108G × N/A	72.0	90.9
MLDC_8f_	49G × 10	74.2	91.5
MLDC_16f_	99G × 10	75.4	92.8
MLDC_EDT − 16f_	75G × 10	**75.6**	92.0

The bold values in each column are all the numbers with the largest percentages.

Compared with other algorithms, the MLDCEDT algorithm optimized by EDT technology has obvious computational advantages.

#### Performance comparison with the current optimal algorithm

The comparison results of the MLDC algorithm and the current optimal algorithm are shown in Table 5.

**Table 5:** Comparison of the MLDC algorithm with the current optimal algorithm.

**Method**	**Backbone network**	**#frame**	**FLOPs × views**	**Top-1 (%)**	**Top-5 (%)**
TSN	InceptionV3	3	3.2G × 250	72.2	90.7
ARTNet	Resnet18	16	23.5G × 250	70.3	89.4
S3D-G	InceptionV1	64	71.4G × 30	74.2	**93.6**
I3D	InceptionV1	64	108G × N/A	72.4	90.2
R (2+1) D	ResNet-34	32	152G × 10	74.2	91.4
TSN (our impl.)	ResNet-50	8	33G × 10	69.5	89.8
C2D	ResNet-50	32	N/A	71.7	89.9
NL C2D	ResNet-50	32	N/A	74.2	91.2
NL I3D	ResNet-50	32	N/A	74.1	91.4
TSM	ResNet-50	16	65G × 30	74.6	91.6
SlowFast	ResNet-50	4+32	75.6	75.4	92.3
CoST	ResNet-101	8	N/A	**75.7**	92.7
STM	Resnet-50	16	67G × 10	73.2	91.5
CPNet	Resnet-101	32	N/A	75.0	92.2
(ours) MLDC	Resnet-50	16	99G × 10	75.3	92.1

The bold values in each column are all the numbers with the largest percentages.

In 2D networks, MLDC outperforms NLResnet-50 by 1.4%, TSM by 2%, and STM by 2%. Compared with the algorithm of 3D network, the MLDC algorithm still exceeds the recognition accuracy of the S3D-G model by 2% and the I3D network by 3.6% even with fewer inputs. The experimental results show that the MLDC algorithm is very competitive with the current SOTA algorithm.

#### Comparison test on something-something V1 data set

In this paper, the MLDCTSM is used to represent the fusion network of MLDC algorithm and TSM. The experimental results are shown in Table 6.

**Table 6:** Performance comparison of MLDC on something-something V1 dataset.

**Method**	**Backbone network**	**#FLOPs**	**#parameters**	**Top-1**	**Top-5**
TSN	Inception	16G	10.7M	19.7%	–
TRN-multiscale	BN inception	33G	18.7M	34.2%	–
ECO	BN inception+3D ResNet18	32G	47.5M	39.0%	–
Non-local I3D with GCN	ResNet-50	303G × 2	62.2M	46.3%	76.1%
TSM	ResNet-50	33G	24.3M	45.9%	74.2%
MLDC_TSM_	ResNet-50	37G	24.3M	**47.4%**	**76.6%**

Compared with the TSM network, the MLDCTSM network improves the classification accuracy by 1.4% while keeping the total amount of network computation almost unchanged (<2%). Compared with other current major algorithms, the performance of the MLDCTSM network significantly outperforms the 2D convolution-based network (e.g., 15.7% over the TRM-Multiscale network) and the 3D convolution-based network (e.g., 0.9% over the Non-local I3D network). Therefore, it is a high-performance and low-loss solution to improve the timing modeling capability of the network by embedding MLDC modules.

#### Analysis of reconstruction time

This paper evaluates the reconstruction time between the contrast algorithms. Table 7 shows the time complexity (in seconds) required by each algorithm to reconstruct the six videos in the common data set.

**Table 7:** Comparison of reconstruction time of different algorithms.

**Network model**	**Rebuild time (/s)**	**Frame rate (FPS)**
TSN	183.65	0.67
TRN-Multiscale	72.18	0.89
ECO	45.85	0.58
Non-local I3D with GCN	169.37	2.78
TSM	341.28	6.86
Algorithm	25.49	10.71

The minimum temporal complexity value and the maximum frame rate in these algorithms are marked in bold. In the table, except for the algorithm in this paper, other algorithms are based on iterative optimization, and the running time of such algorithms is directly related to the complexity of each iteration. Because each iteration needs to search for non-local similar patches and ensure the minimization of the weighted kernel function, it takes 1 hour for TSN to reconstruct 8 frames from a single compressed measurement. However, the algorithm proposed in this paper only needs to input the compressed measurement frame into the whole network, and the reconstruction result can be obtained through the feed-forward calculation of the neural network, thus realizing nearly real-time reconstruction.

## Discussion

**Figure 5:**
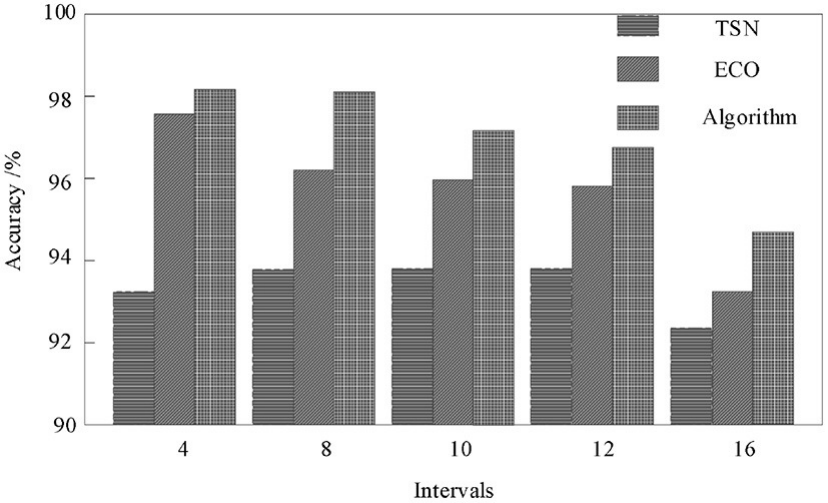
Comparison of test results.

For the action analogy with obvious semantic features, such as Basketball, Fencing, Horse Riding, etc., the network based on multi-modal data fusion can be improved to some extent. But for the action categories such as Skate Boarding, ski jet, surfing, etc., when their semantic features are relatively vague, by adding the skeleton information, the recognition accuracy of the model has been significantly improved. The effectiveness of the method proposed in this paper can be confirmed by comparing the analytical experimental results in Figure 5.

When the interval frame number is 4, the best result-98.8% can be achieved. When the interval frame number is <12, the accuracy of the result is maintained at more than 95%. When the number of interval frames is 16, the accuracy is significantly reduced.

## Conclusion

Based on the video feature representation algorithm based on dependency capture, this paper analyzes the problems of this kind of algorithm, such as inaccurate modeling dependency relationship, a large amount of calculation, and insufficient utilization of neighborhood pixel information, and proposes a multi-semantic long-range dependency capture algorithm to enhance the long-range dependency capture ability of the convolutional neural network.

(1) The multi-semantic long-range dependency capture module is divided into three steps from the algorithm level: pixel aggregation, semantic unit correlation calculation, and dependency establishment. The algorithm process of each step is described respectively. It is proposed to form semantic units with richer semantic information by introducing more pixels in the dependency modeling process and to model the dependencies between semantic units.
(2) In the experimental setting, we introduce two large-scale data sets in the video understanding direction: Kinetics and Something-Something V1, and introduce the optimization strategies and reasoning methods used by the network.
(3) The algorithm is tested on the Kinetics data set to explore the optimal configuration of the network. The MLDC algorithm is tested on the Something-Something V1 data set for fine motion classification, which proves that the MLDC algorithm also has significant advantages in temporal motion modeling.

The algorithm proposed in this paper shortens the reconstruction time and improves the reconstruction quality, but the number of parameters in the overall network is too large, which requires a lot of GPU computing power. As a result, the algorithm proposed in this paper cannot be directly deployed to mobile terminals, which limits the practical application of imaging systems based on compressed sensing. Future studies should consider using pruning operations or redesigning lightweight modules to reduce the parameters in the network.

## Data availability statement

The raw data supporting the conclusions of this article will be made available by the authors, without undue reservation.

## Author contributions

The author confirms being the sole contributor of this work and has approved it for publication.

## Conflict of interest

The author declares that the research was conducted in the absence of any commercial or financial relationships that could be construed as a potential conflict of interest.

## Publisher's note

All claims expressed in this article are solely those of the authors and do not necessarily represent those of their affiliated organizations, or those of the publisher, the editors and the reviewers. Any product that may be evaluated in this article, or claim that may be made by its manufacturer, is not guaranteed or endorsed by the publisher.
